# ASSOCIATION BETWEEN PROVIDER DISCONTINUITY AND QUALITY OF CARE FOR NURSING HOME RESIDENTS

**DOI:** 10.1093/geroni/igad104.2087

**Published:** 2023-12-21

**Authors:** Hyunkyung Yun, Mark Unruh, Hye-Young Jung

**Affiliations:** Brown University, Providence, Rhode Island, United States; Weill Cornell Medical College, Ithaca, New York, United States; Weill Cornell Medical College, New York City, New York, United States

## Abstract

Discontinuity in outpatient care has been associated with lower quality care in community settings, but little is known about discontinuity of care in nursing homes (NHs). We examined discontinuity in clinician care and care quality for NH residents using Medicare fee-for-service claims for a 20% national sample of beneficiaries (January 1, 2012 to December 31, 2019). For residents with NH stays of ≥2 years, we examined changes in attributed clinicians during the first two years of their stays. Residents were attributed to the physician or advanced practitioner with a plurality of visits provided to them in the NH in a given year. Estimates were adjusted for resident, clinician, and NH characteristics. The determinant of interest reflected whether there was a change in the attributed clinician from the first to the second year. Among 280,831 residents in our sample, 37.6% (105,551) were attributed to a different clinician in their second year. On a quarterly basis, these residents had a 0.2 percentage point [pp] (95%CI, 0.2-0.3; p-value <.001) higher likelihood of an ambulatory care sensitive (ACS) hospitalization, a 0.2 pp (95% CI, 0.1-0.3; p-value <.001) higher likelihood of an ACS emergency department (ED) visit, and $609 (95% CI, $548.4-$669.5; p-value <.001) higher health care costs in their second year compared to residents without a clinician change. Compared to baseline rates, these represent relative increases of 14.2% in ACS hospitalizations, 11.5% in ACS ED visits, and 8% in costs. Discontinuity in care is associated with lower-quality care for NH residents.

